# Health behavior and medical insurance under the healthy China strategy: a moral hazard perspective

**DOI:** 10.3389/fpubh.2024.1315153

**Published:** 2024-12-13

**Authors:** Linhong Chen, Lingyu Zhang, Xiaocang Xu

**Affiliations:** ^1^School of Marxism, Chongqing Technology and Business University, Chongqing, China; ^2^School of Economics and Management, Huzhou University, Huzhou, China

**Keywords:** healthy China strategy, psychological deviation, moral hazard, medical insurance, out-of-pocket medical expenditure

## Abstract

**Introduction:**

High medical expenditure is one of the major obstacles to achieving common prosperity in China. As a health risk compensation and protection mechanism, medical insurance has played a good role in alleviating the economic burden of patients. However, due to the existence of moral hazard, medical insurance may also lead to the occurrence of psychological deviation and overtreatment of patients or hospitals’ health treatment expectations, thus generating unnecessary pressure on public financial expenditure.

**Methods:**

Using the China Health and Retirement Longitudinal Survey (CHARLS) and Heckman model, this paper discusses the difference in the impact of medical insurance on outpatient and hospitalization costs. The change of the proportion of out-of-pocket medical expenditure is further analyzed.

**Results:**

The study found that while medical insurance reduced the probability of outpatient visits and increased the probability of hospitalization, it increased the cost of outpatient visits and hospitalization. Further, it reduces the share of out-of-pocket medical expenditure.

**Discussion:**

This shows that medical insurance does play a role in alleviating the financial pressure of patients, but the overtreatment caused by moral hazard cannot be ignored, especially the over-examination and over-prescribing of drugs in the outpatient process.

## 1 Introduction

The problem of the aging population continues to worsen in China. According to the Bulletin of the Seventh National Census (2021), 190.6 million people aged 65 and above in China, accounting for 13.50% of the total population (National Bureau of Statistics, 2021). The problem of health problems and health expenditure is more prominent. First, cardiovascular disease, diabetes, and other chronic health problems are uncertain (1). According to the World Health Statistics Report 2021 released by the World Health Organization (WHO), the premature death rate from non-communicable diseases (NCDS) among Chinese residents aged 30–70 was 15.9 percent in 2019. Secondly, the rapid growth of medical and health expenditure has put tremendous pressure on family life and social and economic development. Between 2011 and 2018, 19.7 percent and 5.4 percent of Chinese households spent more than 10 percent and 25 percent of their total household income on health, according to who data. As a disease risk compensation mechanism, medical insurance can reduce the economic burden of medical treatment. Some scholars believe that medical insurance reduces the threshold of medical use, improves the utilization rate of medical and health services, satisfies the potential medical consumption demand, and may also increase medical expenditure due to excessive medical treatment (2, 3). Some other scholars believe that the impact of medical insurance shows group differences and heterogeneity for different income groups (4).

The medical insurance system has been the focus of scholars around the world, and most scholars hold a positive attitude toward the medical insurance system. Zhu (5) and Xu and Wang (6) proposed medical insurance could disperse the risk of disease. Some scholars believe that the subsidy effect of medical insurance on medical consumption reduces the threshold of medical and health services (2, 7, 8). Chinese Longitudinal Healthy Longevity Survey (CLHLS) was used to test the impact of medical insurance on the medical services demand of the older adult in China (9–11). He et al. (2016) used logistic regression found that medical insurance can significantly improve the utilization rate of medical and health services in 2011 and 2013 (12–14). In addition, many scholars believe that medical insurance can also improve residents’ health status and contribute to the improvement of resident’s health level (15–17), which is conducive to reducing residents’ medical expenses in the long run.

However, some scholars deny the positive effect of medical insurance on health behavior. Wang (18) found that compared with those who did not use inpatient services, the incidence of catastrophic health expenditure among urban employees insured by medical insurance increased by 2.25 times from 2011 to 2015, indicating an unreasonable increase in medical expenses. Due to the existence of moral hazard, adverse selection, and other factors, the positive role of medical insurance is greatly reduced. Ren (19) empirically tested whether the adverse selection and moral hazard exist in basic medical insurance by using Heckman model and Logit model, and concluded that adverse selection exists in basic medical insurance. Older persons in poor health are more likely to participate in basic health insurance; The probability of participating in physical exercise among the older adult with basic medical insurance is significantly reduced, which to some extent verifies the existence of moral hazard. Li and Yu (20) also believes that moral hazard and information asymmetry positively affect total medical expenses. Medical insurance different starting line and increased subsidies for taking medical consumables kickbacks medical staff left a profit space, to a certain extent caused the excessive medical treatment health care services such as abuse, weaken the welfare effect of medical insurance, and even the medical expenditure shows a tendency of not reasonable growth, Yang (21) argue that given the current medical service charge payment mechanism, The new Rural cooperative medical scheme is also likely to drive up medical costs and steer patients toward more expensive and unnecessary hospital care.

In addition, some scholars believe that the impact of medical insurance on health behavior has group heterogeneity in different income groups and different household registration types. Therefore, the fairness of the medical insurance system needs to be improved. Alkenbrack (22) believes that community health insurance provides richer people with more protection than the poor. Similarly, Chen (23) believes that new rural cooperative medical care will reduce the out-of-pocket medical expenditure of the rich more significantly in China, and rural families need higher generosity (24). Jin and Yu (25) also proposes that the medical insurance system does not generally adjust income redistribution. Yao (26) believes that there are significant differences between urban and rural areas in access to medical care and utilization of medical resources among respondents who also have medical insurance. In terms of medical motivation and medical cost burden, the positive effect of medical insurance on rural respondents is much lower than that of urban Hukou respondents. On the contrary, the “reverse redistribution” phenomenon exists in which low-income groups subsidize high-income groups.

To sum up, the impact of medical insurance on health behavior is still controversial in the current research results, which will bring confusion to the government in the specific implementation process of medical insurance system. More importantly, China’s basic medical insurance system adopts a model that combines social pooling with individual accounts. The pooling fund and individual account bear different responsibilities for paying medical expenses, and there are differences in the reimbursement ratios and thresholds for outpatient and inpatient expenses. The pooling fund is mainly used to pay for hospitalization expenses, and the proportion of medical insurance reimbursement for hospitalization is relatively high. The medical expenses of Outpatient department services can only be paid through personal accounts. In the eyes of the public, personal accounts are equivalent to their own private money. Therefore, the marginal contribution of this article is to discuss the differences in the impact of medical insurance on health behavior based on the latest data released by CHARLS.

## 2 Methods

### 2.1 Variable selection and data preprocessing

The Andersen’s behavioral model of health services use (BMHSU), was constructed by Ronald Andersen (27), is to explain why individuals/families use health services, measure the equitable accessibility of health services, and analyze the influencing factors of individual medical behavior. Anderson model is composed of three factors: Predisposing characteristics, Enabling resources, and Need factors, each of which includes its own variables. Anderson model can provide a complete theoretical framework for researchers to systematically analyze individual medical behavior and help decision-makers improve the utilization of medical services through the reform of the medical and health system. After a large number of empirical studies, the Anderson model has been recognized as the authoritative medical service research model in the field of international health service research in the past 50 years (28).

#### 2.1.1 Explained variable

This paper tries to understand health behavior from three aspects: outpatient, hospitalization, and the proportion of out-of-pocket expenditure in total expenditure. Out-patient expenditure refers to the medical treatment out-patient expenditure in the past year; Hospitalization costs is the total hospitalization costs for the most recent visit within the past year; Total medical expenditure is the sum of the most recent outpatient costs and hospitalization costs in the past year. Proportion of out-of-pocket medical expenditure is the ratio of out-of-pocket medical expenditure to total medical expenditure.

#### 2.1.2 Core explanatory variable

To explore the impact of medical insurance on medical expenses of middle-aged and older adult people, the main explanatory variable selected in this paper is medical insurance characteristics, which is specifically reflected in the questionnaire “Do you currently participate in the following medical insurance?.” It includes medical insurance for urban employees, urban and rural residents, urban residents, new rural cooperative medical insurance, free medical care, medical assistance, commercial medical insurance, long-term care insurance, and other medical insurance. In this study, participating in medical insurance was defined as 1, and not participating in medical insurance was defined as 0.

#### 2.1.3 Other explanatory variables

Whether an individual has medical expenses is affected by the characteristics of medical insurance and affected by many other factors. In selecting other explanatory variables, this study referred to the Anderson model of medical and health service utilization and selected explanatory variables from three aspects: tendency characteristics, enablement factors, and demand.

##### 2.1.3.1 Predisposing characteristics

###### 2.1.3.1.1 Gender

Male is 1, female is 0.

###### 2.1.3.1.2 Age

The actual birth age shall prevail, and the actual birth year shall be adopted to calculate the age of the respondents according to the data survey year 2018 as the cut-off point, and unreasonable variables under 45 years old shall be excluded.

###### 2.1.3.1.3 Education level

Education level is divided into four categories according to the respondents’ highest level of education (excluding adult education): 0 indicates that they have not received formal education; (1) Have received primary education or below (including private schools, kindergartens, pre-schools and primary schools); (2) Have received education above primary school, secondary school or below; (3) College education or above.

###### 2.1.3.1.4 Residential address type

Based on the current residence, the value of main urban area/urban–rural integration department/town center/township combination department is 0, and the value of rural central area/village and special area is 1.

###### 2.1.3.1.5 Marital status

Unmarried (never married, cohabitation), divorced (no longer separated as a spouse, divorced, widowed) is 0, married is 1.

##### 2.1.3.2 Enabling resources

###### 2.1.3.2.1 Access to health resources

The number of kilometers traveled from the respondent’s home to a medical facility receiving outpatient or inpatient medical services was reflected in the number of kilometers.

###### 2.1.3.2.2 Total annual household income

It mainly includes wage income and transfer payments received by all members of the family, household agricultural income (including income from agricultural products, livestock and aquatic products), income from self-employed or private enterprises, and household public transfer payment income (including subsistence allowance income and social/state donation income). In order to deal with the skewness distribution of annual household total income data, the logarithmic correction of annual household total income was conducted in this study.

##### 2.1.3.3 Need factors

###### 2.1.3.3.1 Self-rated health status

Self-rated health status comes from the subjective evaluation of the health status of the respondents in the CHARLS database, and there are five measures for the respondents to evaluate themselves: very good, good, fair, poor and very poor. In this data processing, the self-rated health status is assigned 0 for Very poor and poor, 1 for fair and 2 for good and Very Good.

###### 2.1.3.3.2 ADL scale

ADL scale was used to examine the physical function of the subjects from six aspects of walking, dressing, eating, bathing, going to the toilet and getting on and off the bed. Among them, “walking” mainly tests whether the subjects can walk 100 meters by themselves; “Dressing up” reflects the difficulty of taking clothes out of the closet, putting them on, buttoning them up and belting them up. “Eating” tested whether the subjects could eat independently. “Going to the toilet” mainly tests whether there is difficulty in relieving oneself, including squatting and standing; “Bath” includes a shower or bath. 1 if you have difficulty with at least one of these behaviors for health and memory reasons, 0 otherwise.

### 2.2 Model construction

The medical expenditure data used in this study presented a skewed distribution. The vast majority of individuals had zero medical expenditure in the past month; there is a significant gap in the amount of medical expenditure between individuals who have medical expenditure in the past month. Among them, the medical expenditure of the investigated individual in the past month is 0, which may be in the following situations: First, the investigated individual is in good health in the past month and has no medical consumption demand, so the medical expenditure in the past month is 0; Second, the respondents had medical consumption demand in the past month, but subjectively chose to give up medical treatment due to economic pressure and accessibility of medical resources, resulting in 0 medical expenditure in the past month. Combined with the above situation, for such skewness distribution data, if OLS regression is performed directly, it will often lead to selection bias and other endogenous problems.

Therefore, this paper uses the Two-part model and Heckman sample selection model to avoid the endogeneity of sample selection error. The steps are shown in Table 1.

**Table 1:** Detailed steps of model regression.

Steps	Two-part model		Heckman model
	Probability model	Expenditure model	
**1**	With the probit model to estimate the probability of the individuals choose to use the health services $P_{i}\left(y_{i}>0|x_{i}\right)$	Estimated medical expenditure of individuals with a positive probability of using health services $E\left(y_{i}|x_{i}|y_{i}>0\right)$	The probit model is used to estimate whether patients choose to go to the doctor and incur medical expenses: $P_{i}=\{\begin{array}{c} 1，x_{i}\beta_{1}+\varepsilon_{1i}>0\\ 0，x_{i}\beta_{1}+\varepsilon_{1i}\leq 0 \end{array}$ Where, $P_{i}$ =“0” represents medical expenditure without medical treatment; $P_{i}$ =“1” represents medical expenditure with medical treatment.
**2**		The least square method was used to estimate individual medical expenditures with a positive probability of health service	The result equation is used to estimate the individual medical expenditure of the existing medical treatment behavior as follows: $\log\left(y_{i}|p_{i}=1\right)=x_{i}\beta_{2}+\varepsilon_{2i}$
**3**		The final estimate of individual medical expenditure is obtained by multiplying the estimated probability of visit by the estimated individual medical expenditure $E\left(\mathrm{logy}|x\right)=P_{r}\left(y>0|x\right)E\left(\mathrm{logy}|,y>0|,x\right)$	

In addition, in order to explore the impact of medical insurance on the medical burden of middle-aged and older adult groups, in the empirical analysis of the impact of medical insurance on out-of-pocket medical expenditure, this paper uses the relative value of total medical expenditure (that is, the proportion of out-of-pocket medical expenditure in total medical expenditure), and uses the generalized linear model (GLM) to estimate. STATA 16 is used for all data processing in this paper.

### 2.3 Data sources

The data of this study is from the latest phase of China Health and Retirement Longitudinal Survey (CHARLS, 2018). CHARLS is a large-scale interdisciplinary survey conducted by Peking University based on the international experience of advanced countries in the research field, including the Health and Retirement Survey (HRS) of the United States, the older adult Follow-up Survey (ELSA) of the United Kingdom and the Health, Aging and Retirement Survey (SHARE) of Europe. PPS sampling method is adopted in county/district sampling stage, and CHALRS-GIS technology is used as sampling frame at village level. This paper collected 19,000 respondents from 12,400 households in 450 communities (villages) covering 28 provinces, which can scientifically and fairly represent the high-quality microdata of families and individuals aged 45 and above in China.

In addition, all the currencies mentioned in this paper are Chinese yuan.

## 3 Results

### 3.1 Descriptive analysis of samples

In the survey data sorted out, there were 12,248 valid sample records, and 3,913 sample records of medical expenditure within 1 month of the survey time.

As shown in Table 2, in the latest utilization of medical and health services in the past year, all samples’ average total medical expenditure was 2461.272 yuan, the average out-patient expenditure was 245.718 yuan, and the average inpatient expenditure was 2215.554 yuan. The vast majority of the sample was covered by medical insurance.

**Table 2:** Sample descriptive statistics.

Variable name	Variable definitions	Mean	Standard deviation
ME	Total medical expenditure	2461.272	16869.345
OC	Outpatient costs	245.718	3357.929
HC	Hospitalization costs	2215.554	16429.228
OPME	Out-of-pocket medical expenditure	4145.809	19083.38
Insurance	Whether or not to participate in medical insurance	0.969	0.173
Gender	gender	0.415	0.493
Age	age	62.903	10.026
Address	Residential address type	0.74	0.438
Education	Education Level	1.054	0.768
Marital	Marital status	0.84	0.367
Distance	Access to medical resources	31.511	147.71
House income	Total annual household income	32506.541	80816.835
Loghi	Log (Total annual household income)	9.422	1.512
Selfh	Self-rated health status	0.857	0.712
ADL	ADL scale	0.345	0.475

In terms of tendency characteristics, the average age of all samples was 62.903 years old, and the number of male samples was larger than that of female samples. The majority of the respondents are rural residents, and the education level is concentrated in the middle school stage. A relatively small number of respondents are unmarried. In terms of enabled resources, the average distance between the respondent’s family and the medical institution receiving medical and health services is 31.511 km, and the average annual household income is 32506.541 yuan, and the average annual household income after logarithmic treatment is 9.422 yuan. In terms of demand, the mean value of self-rated health status of the respondents was 0.857, indicating that the health status of the samples was in the middle and lower level, which may be related to the older age of the samples. The mean value of ADL scale was 0.345, indicating that the overall self-care ability of the samples was good.

According to the interactive analysis results (Table 3), the average medical expenditure of middle-aged and older adult people who participate in medical insurance are higher than those who do not participate in medical insurance, and medical insurance does not play a substantial subsidy role. In out-patient expenditure, the average out-patient expenditure of middle-aged and old people with medical insurance was 247.77 yuan, the average out-patient expenditure of middle-aged and old people without medical insurance was 181.66 yuan, the average out-patient expenditure gap was 66.11 yuan; In the hospitalization costs, the average hospitalization costs of the middle-aged and older adult group with medical insurance was 2245.73 yuan, 972.52 yuan more than the average hospitalization costs of 1273.21 yuan without medical insurance. The average total medical expenditure of the middle-aged and older adult group with medical insurance (2493.50) is 1038.63 yuan higher than that of the group without medical insurance (1454.87). Due to the compensation effect of medical insurance, this gap is narrowed in the total medical expenditure, and the average total medical expenditure of the middle-aged and older adult group with medical insurance (4151.04) is 253.64 yuan higher than that of the group without medical insurance (3897.40).

**Table 3:** Interactive analysis results.

**variables**	**Whether or not to participate in medical insurance**		**Pearson chi**
	**YES**	**NO**	
Outpatient costs	247.77	181.66	300.54***
Hospitalization costs	2245.73	1273.21	163.71***
Total medical expenditure	2493.50	1454.87	165.21***
Out-of-pocket medical expenditure	4151.04	3897.40	41.38***

***p < 0.01, **p < 0.05, *p < 0.1.

### 3.2 Outpatient costs and hospitalization costs

#### 3.2.1 Impact on outpatient costs

Table 4 shows the empirical results of the two-part model and Heckman model of medical insurance for the out-patient expenditure of middle-aged and older adult groups.

**Table 4:** Influence of medical insurance on outpatient costs.

Variable	Two-part model		Heckman model	
	Outpatients probability model	Outpatient expenditure model	Selection model	Expenditure model
Participating in medical insurance (control group: uninsured)	−0.289* (0.152)	0.403* (0.226)	−0.292* (0.152)	0.435* (0.229)
Female (control group: male)	−0.201*** (0.046)	0.217*** (0.076)	−0.201*** (0.046)	0.242*** (0.081)
Age	−0.024*** (0.002)	0.007* (0.004)	−0.024*** (0.002)	0.010* (0.005)
Rural (control group: urban)	0.092* (0.051)	−0.409*** (0.085)	0.093* (0.051)	−0.420*** (0.086)
Education level (control group: no formal education)				
Primary and below	0.006 (0.055)	0.013 (0.092)	0.007 (0.055)	0.012 (0.092)
Above primary school, secondary school and below	0.086 (0.066)	0.192* (0.109)	0.086 (0.066)	0.181* (0.110)
Junior college or above	0.341** (0.170)	−0.031 (0.271)	0.341** (0.170)	−0.072 (0.275)
Married (control group: non-married)	−0.004 (0.059)	0.254** (0.100)	−0.003 (0.059)	0.254** (0.010)
Distance from hospital	−0.003*** (0.000)	0.007*** (0.001)	−0.003*** (0.000)	0.007*** (0.001)
Log (Annual household income)	0.019 (0.015)	−0.016 (0.024)	0.019 (0.015)	−0.018 (0.025)
Self-rated health status (control group: poor)				
General	0.049 (0.046)	−0.241*** (0.076)	0.049 (0.046)	−0.246*** (0.076)
Good	−0.082 (0.076)	−0.440*** (0.128)	−0.080 (0.076)	−0.429*** (0.129)
Difficulty in daily activities (control group: no difficulty in daily activities)	−0.036 (0.047)	0.061 (0.076)	−0.035 (0.047)	0.066 (0.078)
Constant	1.780*** (0.284)	4.897*** (0.448)	1.783*** (0.284)	4.846*** (0.452)

***p < 0.01, **p < 0.05, *p < 0.1; (): standard error.

Medical insurance has a negative effect on the probability of outpatient visit, but has a positive effect on the expenditure of outpatient visit, and the results are significant at 10% confidence level. Female have lower outpatient attendance rates than Male, but higher outpatient costs. Rural residents have a higher probability of outpatient treatment than urban residents, but the outpatient costs is lower. According to the level of education, the higher the level of education, the higher the probability of outpatient visits, the realization of outpatient expenses first increases and then decreases, but only a few education level is significant. Among marital status, married individuals showed lower (but statistically insignificant) rates of outpatient visits and higher outpatient costs (significant confidence level of 5%) compared to unmarried individuals. In the 1% confidence interval, the distance to the hospital has a negative impact on the probability of outpatient visits and a positive impact on outpatient costs. Respondents with fair and good self-rated health had lower outpatient costs (significant at the 1% confidence level) than those with poor self-rated health. The empirical results of the two-part model are basically consistent with the Heckman model.

#### 3.2.2 Impact on hospitalization costs

Table 5 shows the empirical results of the two-part model and Heckman model of medical insurance’s hospitalization costs for middle-aged and older adult groups.

**Table 5:** Impact of medical insurance on hospitalization costs.

Variable	Two-part model		Heckman model	
	Hospitalization probability model	Hospitalization costs model	Selection model	Expenditure model
Participating in medical insurance (control group: uninsured)	0.419*** (0.150)	0.047 (0.203)	0.378** (0.149)	−0.175 (0.219)
Female (control group: male)	0.192*** (0.047)	0.076 (0.056)	0.191*** (0.046)	−0.028 (0.061)
Age	0.025*** (0.002)	0.003 (0.003)	0.024*** (0.002)	−0.011*** (0.003)
Rural (control group: urban)	−0.132** (0.052)	−0.379*** (0.063)	−0.106** (0.052)	−0.309*** (0.069)
Education level (control group: no formal education)				
Primary and below	−0.047 (0.056)	0.012 (0.066)	−0.049 (0.055)	0.039 (0.072)
Above primary school, secondary school and below	−0.007 (0.067)	0.240*** (0.081)	−0.033 (0.067)	0.237*** (0.088)
Junior college or above	−0.382** (0.169)	0.325 (0.221)	−0.398** (0.168)	0.562** (0.239)
Married (control group: non-married)	0.039 (0.060)	0.087 (0.070)	0.036 (0.060)	0.059 (0.078)
Distance from hospital	0.003*** (0.000)	0.001*** (0.000)	0.003*** (0.000)	0.000** (0.000)
Log (Annual household income)	−0.023 (0.015)	0.048*** (0.018)	−0.020 (0.015)	0.060*** (0.020)
Self-rated health status (control group: poor)				
General	−0.220*** (0.047)	−0.073 (0.057)	−0.221*** (0.047)	0.057 (0.063)
Good	−0.276*** (0.077)	−0.102 (0.100)	−0.268*** (0.076)	0.059 (0.107)
Difficulty in daily activities (control group: no difficulty in daily activities)	0.113** (0.048)	0.141** (0.057)	0.119** (0.047)	0.079 (0.062)
Constant	−1.498*** (0.286)	7.871*** (0.358)	−1.462*** (0.283)	9.507*** (0.405)

***p < 0.01, **p < 0.05, *p < 0.1; (): standard error.

In the two-part model, at the significance level of 1%, the probability of hospitalization increased by 41.9% compared with the group not participating in medical insurance. The probability of hospitalization for females was 19.2% higher than that for males. Age had a positive effect on the probability of hospitalization, and the probability of hospitalization increased with age. Compared with urban residents, rural residents had a significant 13.2% reduction in hospitalization probability (5% confidence level) and 37.9% reduction in hospitalization costs (1% confidence level). With the increase of education level, the probability of hospitalization gradually decreased, and the hospitalization costs gradually increased, but the regression results were only significant in the individual education stage. Marital status has no significant effect on hospitalization costs of middle-aged and older adult groups. There is a significant positive correlation between distance from hospital and hospitalization probability and hospitalization costs, but the influence coefficient is small. Annual household income positively influences hospitalization costs at the confidence level of 1%. The higher the self-rated health status, the lower the probability of hospitalization; the group with difficulty in daily activities has a higher probability of hospitalization and higher hospitalization costs.

In the sample selection model, the partial results of the selection model are basically consistent with those of the two models, with only slight coefficient differences. In the expenditure model, the sample selection model assumes that for every 1-year increase in the age of the middle-aged and older adult population, the medical expenditure decreases by 1.1% (significant at the 1% confidence level).

### 3.3 The proportion of out-of-pocket medical expenditure to total medical expenditure

As shown in Table 6, the relative value of out-of-pocket medical expenditure (the proportion of out-of-pocket medical expenditure to total medical expenditure) is used to evaluate the influence of medical insurance on the out-of-pocket medical expenditure of middle-aged and older adult groups.

**Table 6:** Result of the proportion of out-of-pocket medical expenditure to total medical expenditure.

Variable	dy/dx	Variable	dy/dx
Participating in medical insurance (control group: uninsured)	−0.205*** (0.057)	Married (control group: non-married)	−0.023 (0.023)
Female (control group: male)	−0.034** (0.017)	Distance from hospital	0.000 (0.000)
Age	−0.005*** (0.000)	Log (Annual household income)	−0.005 (0.006)
Rural (control group: urban)	0.044** (0.019)	Self-rated health status (control group: poor)	
Education level (control group: no formal education)		general	0.017 (0.018)
Primary and below	0.010 (0.021)	good	0.029 (0.029)
Above primary school, secondary school and below	−0.062** (0.025)	Difficulty in daily activities (control group: no difficulty in daily activities)	0.008 (0.018)
Junior college or above	−0.073 (0.065)	Constant	1.277*** (0.106)

***p < 0.01, **p < 0.05, *p < 0.1; (): standard error.

According to the proportion model of out-of-pocket medical expenditure, participation in medical insurance reduces the relative value of out-of-pocket medical expenditure by 20.5% on average (*p* = 0. 057). The relative value of out-of-pocket medical expenditures for females was 3.4% less than that for males (*p* = 0.017), and the relative value of out-of-pocket medical expenditures decreased by 0.5% for each 1-year increase in age (*p* = 0.000). According to the average total medical expenditure of 2461.272 yuan in this study, the out-of-pocket medical expenditure of middle-aged and old people participating in medical insurance will decrease by 504.56 yuan.

### 3.4 Robustness test

Table 7 uses the total medical expenditure of middle aged and older adult groups as the dependent variable for robustness testing.

**Table 7:** Influence of medical insurance on total medical expenditure.

Variable	Two-part model		Heckman model	
	Hospitalize probability model	Total medical expenditure model	Selection model	Expenditure model
Participating in medical insurance (control group: uninsured)	0.298 (0.311)	0.614*** (0.227)	0.100 (0.252)	0.552** (0.229)
Female (control group: male)	−0.178 (0.138)	0.382*** (0.070)	−0.256** (0.122)	0.397*** (0.072)
Age	−0.012* (0.007)	0.030*** (0.004)	−0.019*** (0.006)	0.031*** (0.004)
Rural (control group: urban)	−0.098 (0.162)	−0.545*** (0.079)	−0.065 (0.134)	−0.539*** (0.080)
Education level (control group: no formal education)				
Primary and below	0.204 (0.149)	−0.080 (0.084)	0.111 (0.134)	−0.106 (0.086)
Above primary school, secondary school and below	0.416* (0.213)	0.148 (0.101)	0.162 (0.214)	0.104 (0.103)
Junior college or above	−0.187 (0.367)	−0.243 (0.263)	0.098 (0.324)	−0.195 (0.267)
Married (control group: non-married)	0.223 (0.148)	0.162* (0.091)	0.201 (0.129)	0.133 (0.093)
Distance from hospital	0.006* (0.004)	0.002*** (0.000)	0.012*** (0.004)	0.002*** (0.000)
Log (Annual household income)	0.030 (0.045)	−0.008 (0.023)	0.050 (0.039)	−0.011 (0.023)
Self-rated health status (control group: poor)				
General	−0.057 (0.141)	−0.382*** (0.072)	−0.047 (0.124)	−0.375*** (0.073)
Good	−0.282 (0.198)	−0.556*** (0.118)	−0.083 (0.170)	−0.516*** (0.120)
Difficulty in daily activities (control group: no difficulty in daily activities)	0.087 (0.139)	0.271*** (0.072)	0.189 (0.120)	0.262*** (0.073)
Constant	2.256*** (0.778)	5.080*** (0.430)	2.545*** (0.671)	5.162*** (0.438)

***p < 0.01, **p < 0.05, *p < 0.1; (): standard error.

The results of Heckman model are basically consistent, and only the influence coefficients are slightly different. The two-part model showed a 1.2% decrease in the probability of seeing a doctor for each 1-year increase in age at a significance level of 10%. At the significance level of 1%, medical expenses were higher for those with health insurance, women, older age groups, urban residents, and groups with poor self-rated physical condition or difficulty in daily activities; At the significance level of 10%, the married group spent more on health care than the unmarried group.

## 4 Discussion

With China’s aging population becoming an increasingly prominent problem, the explosive growth of health care spending has become a serious problem that needs to be solved, especially in the context of the current Chinese economic situation is not too optimistic. As a health risk compensation and protection mechanism, medical insurance has played a very good role in reducing the economic burden of patients. However, due to the existence of moral hazard, medical insurance may also lead to the occurrence of psychological deviation and overtreatment of patients or hospitals’ health treatment expectations, thus generating unnecessary pressure on public financial expenditure. Using the Chinese Longitudinal Survey on Health and Retirement (CHARLS) and the Heckman model, this paper discusses the difference of the impact of medical insurance on outpatient and hospitalization costs, and further analyzes the change of the proportion of out-of-pocket medical expenditure. We have the following findings:

Firstly, while medical insurance reduced the probability of outpatient visits and increased the probability of hospitalization, it increased the cost of outpatient visits and hospitalization. Further, it reduces the share of out-of-pocket medical expenditure. This shows that medical insurance does play a role in alleviating the financial pressure of patients, but the overtreatment caused by moral hazard cannot be ignored, especially the over-examination and over-prescribing of drugs in the outpatient process (29). The reasons behind this phenomenon lies in: first, from the present situation of the current view, some of the medical staff income linked to drugs, consumables such as sales commissions, it is to guide patients to use more expensive and unnecessary hospital care, and so on the phenomenon of “excessive medical treatment” to provide the right conditions (21), eventually make the older adult group medical expenditure increase; Second, medical insurance benefits medical expenses that residents can enjoy medical treatment service for less medical costs, reduce the threshold of the use of medical services, make the residents can more easily enjoy the medical service, and steadily rising living standards make the residents for their own health more and more attention in China, people are more willing to consumption in medical services. Further, some patients are actively overtreated due to moral hazard.

Secondly, the impact of medical insurance on health behavior has the heterogeneity of gender, age and urban/rural areas. Compared with Male, Female have higher outpatients’ outpatients and higher inpatients’ probability. In general, the total medical expenditure of middle-aged and older adult Female is higher than that of Male, which is different from the conclusion of earlier studies that Male have higher medical expenditure than Female (30, 31). This may be due to improving Female’s social status and economic security ability in recent years. In terms of age, with the increase of age, out-patient expenditure slightly increases, the probability of hospitalization increases, and the total medical expenditure also increases. It may be related to the consumption of health consciousness and health stock. With the increase of age, the health stock is gradually consumed, the probability of disease increases, and the total medical expenditure will also increase. In terms of residential address types, compared with urban residents, rural residents have lower outpatients’ and inpatients’ expenditures, and lower total medical expenditures, which is consistent with the research conclusions of Cheng et al. (32). Both models suggest that married people spend more on outpatient care, which may be related to the degree of health care paid by married people. In terms of enabling factors, the distance to the hospital was directly proportional to the medical expenditure, and the annual household income was directly proportional to the hospitalization costs. In terms of demand, the older adult group with better self-rated health status had lower outpatients or inpatients’ outpatients and corresponding total medical outpatients. Middle-aged and older adult groups with difficulties in daily activities had higher rates of hospitalization costs and higher total medical expenditures.

In a word, the current medical insurance system in China has a certain degree of promoting effect on the medical expenditure of the middle-aged and older adult groups, which will lead to an increase in out-patient expenditure, an increase in the probability of hospitalization, and ultimately an increase in the total medical expenditure. This may be related to the existence of moral hazard, adverse selection and other factors (33), which is an important reason for the blowout growth of China’s medical and health expenditure in recent years.

Therefore, in order to avoid the negative impact of factors such as moral hazard and adverse selection, guarantee health insurance subsidies medical consumption, realize the income redistribution adjustment, first of all, the policy need to standardize medical staff income, guarantee the reasonable income, health care workers to stop drugs such as the phenomenon of medical consumables kickbacks to doctors and nurses, Then, we should strengthen the cultivation of medical ethics of medical staff, improve the moral accomplishment of medical staff, guide medical staff to take appropriate medicine to the case, moderate medication, avoid the waste of medical resources and unnecessary medical burden caused by excessive treatment; At the same time, the medical insurance system needs to improve China’s medical insurance payment mechanism, clear the starting line of payment, refine the medical insurance replenishment policy, give more attention or help to middle-aged and older adult groups with difficulties in daily activities and people with low health evaluation, so that the medical insurance system can better serve the public. Finally, health promotion should be intensified at the macro level, especially for people with bad living habits, such as smoking and drinking, to advocate healthy living and improve national health awareness.

## 5 Conclusion

This paper used the newly published data of CHARLS to verify the relationship between medical insurance and medical expenditure. It is found that medical insurance will increase the outpatients’ expenditure and the probability of hospitalization. Moreover, the total medical expenditure of Female is higher than that of Male in middle-aged and older adult group. In order to avoid the endogenous selection problem in the sample, Two-part models and Heckman model were adopted, but they have been widely used in previous literatures. Therefore, other empirical methods may be tried in future further studies. In addition, other microscopic data such as CFPS and CHIP can also be compared with the results obtained from CHARLS in future studies.

## Data Availability

Publicly available datasets were analyzed in this study. This data can be found here: http://charls.pku.edu.cn/index.html.
